# Comparison of certainty of evidence between the net benefit approach and the traditional GRADE method using the data of Japanese Clinical Practice Guidelines for Management of Sepsis and Septic Shock 2020

**DOI:** 10.1186/s40560-023-00680-5

**Published:** 2023-07-17

**Authors:** Takero Terayama, Hiromu Okano, Sadatoshi Kawakami, Kenichi Kano, Masaaki Sakuraya, Yoshitaka Aoki, Committee of the Japanese Clinical Practice Guidelines for the Management of Sepsis and Septic Shock 2024

**Affiliations:** 1 Department of Emergency, Self-Defense Forces Central Hospital, Ikeziri 1-2-24, Setagaya, Tokyo Japan; 2 Department of Critical Care Medicine, St. Luke’s International Hospital, 9-1 Akashi-Cho, Chuo-Ku, Tokyo 104-8560 Japan; 3 Department of Anesthesiology, Chiba University Graduate School of Medicine, 1-8-1 Inohana, Chuo-Ku, Chiba 260-8670 Japan; 4 Department of Emergency Medicine, Fukui Prefectural Hospital, Yotsui 2-8-1, Fukui City, Fukui 910-8526 Japan; 5 Department of Emergency and Intensive Care Medicine, JA Hiroshima General Hospital, Jigozen 1-3-3, Hatsukaichi, Hiroshima 738-8503 Japan; 6 Department of Anesthesiology and Intensive Care Medicine, Hamamatsu University School of Medicine, Shizuoka, Japan

To the Editor:

Clinical practice guidelines (CPGs) are usually developed using the internationally recognized Grading of Recommendations Assessment, Development, and Evaluation (GRADE) methodology, which requires consideration of the certainty of the estimated treatment effects when making recommendations [1]. Recently, the GRADE Working Group has introduced a novel method, the net benefit approach, which can evaluate the certainty of evidence (CoE) in a more direct and comprehensible manner by utilizing the net effect estimate of significant outcomes to demonstrate the balance of benefits and harms with relative values [2]. In this approach, CoE is determined mainly based on the precision of the net effect estimate. The precision can be considered downgrading by performing a sensitivity analysis that allows the relative value of the outcome that has the most impact on the net effect estimate to vary within a reasonable range. However, to date, there are no officially established CPGs employing the net benefit approach, and no comparative reports exist between this approach and traditional method. Therefore, our study aimed to classify Clinical Questions according to differences of the CoE between the new and the traditional method and to compare characteristics between groups by utilizing CQ data from the Japanese Clinical Practice Guidelines for Management of Sepsis and Septic Shock 2020 (J-SSCG2020), in which CoE was assessed with the traditional GRADE method [3].

Eligible CQs were identified from the J-SSCG2020 data that satisfied the criteria outlined in Table 1. The CoE for each CQ evaluated using the traditional method was obtained from the published data. Two authors independently assessed the CoE of each CQ using the net benefit approach, as illustrated in Additional file 1: Fig. S1. The net benefit approach procedures were semi-automated using an Excel file designed for this study based on the original article [2] (Additional file 2). We classified the CQs into three categories based on changes in the CoE using the net benefit approach as compared to using the traditional method: those with a decrease in the CoE (Group I), those with no change (Group II), and those with an increase (Group III). We have described the characteristics of each group, with continuous variables expressed as medians (interquartile ranges) and categorical variables expressed as numbers with corresponding percentages.

**Table 1:** Inclusion and exclusion criteria to select eligible clinical questions^a^ in this study

Criteria
*Inclusion criteria*
1) Certainty of evidence was evaluated using the GRADE
2) Outcomes to be combined for calculating net effect estimate are dichotomous variables^b^^,c^
*Exclusion criteria*
1) Diagnostic test accuracy or network meta-analysis
2) Only one outcome to be combined for calculating net effect estimate

GRADE, Grading of Recommendations Assessment, Development, and Evaluation

^a^The clinical questions were selected from the Japanese Clinical Practice Guidelines for Management of Sepsis and Septic Shock 2020

^b^The net benefit approach cannot be applied to clinical questions with both dichotomous and continuous outcomes

^c^TT and HO determined the outcomes to be combined to calculate net effect estimate

Figure 1 summarizes the process of selecting CQs. Among the 127 CQs in the J-SSCG2020, 20 were deemed eligible for this study. These were classified as one CQ (5%) in group I, 10 CQs (50%) in group II, and 9 CQs (45%) in group III. The details of these CQs are shown in Additional file 1: Table S1. Table 2 presents the characteristics of the CQs. They were similar except for the number of CQs in which the direction (benefit or harm) of the point estimate in the outcomes. The CoE by the traditional method ranged from “low” to “very low”. Most CQs had outcomes classified as serious or very serious in terms of precision. In addition, their outcomes did not satisfy the optimal information size (OIS). The number of CQs in which the direction (benefit or harm) of the point estimate in the outcomes was consistent was four (40%) in Group II and one (11%) in Group III. Additional file 1: Table S2 presents the patterns of change in CoE between the net benefit approach and the traditional method.

**Fig. 1:**
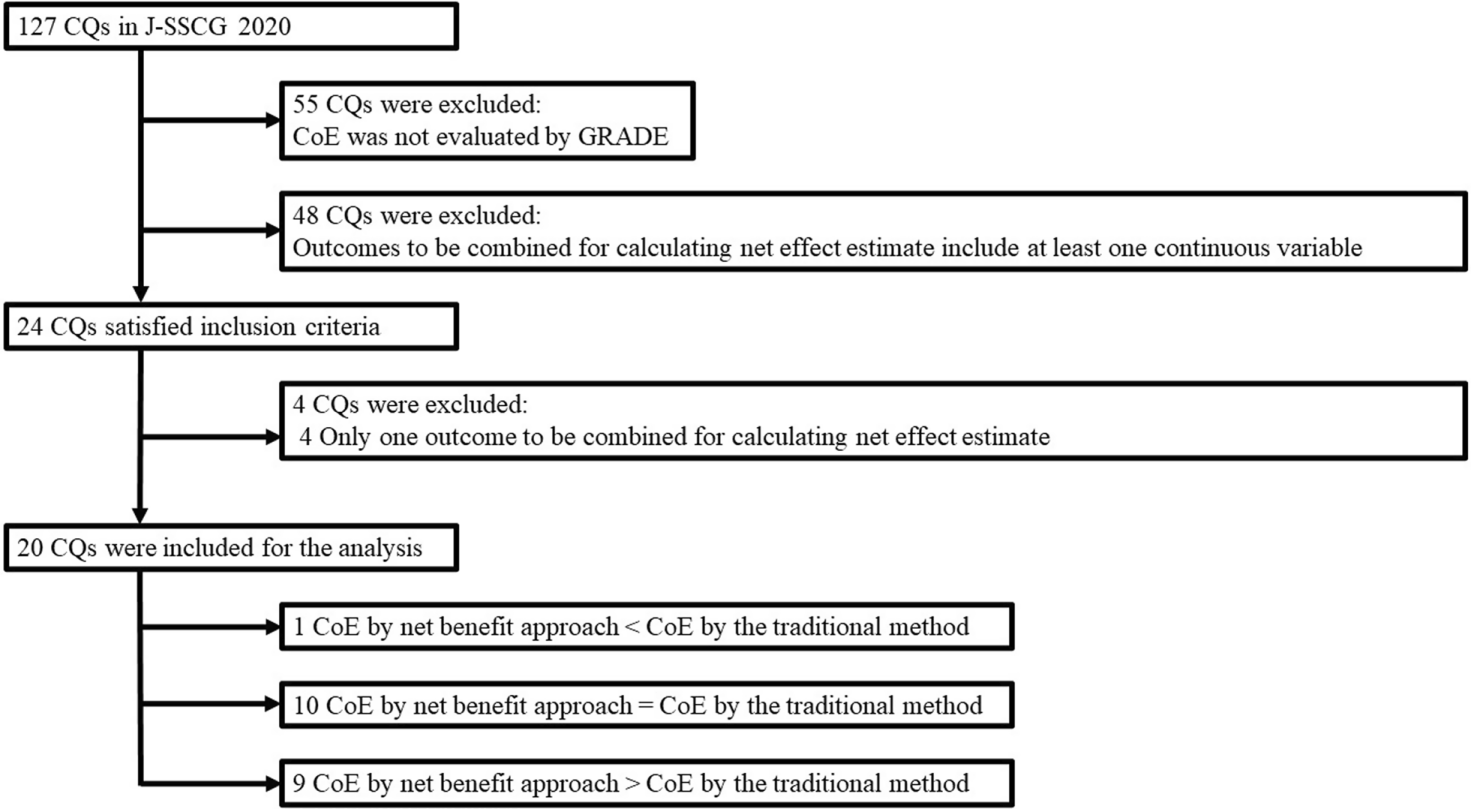
Clinical questions enrollment flowchart. *CQ* clinical question, *J-SSCG 2020* the Japanese Clinical Practice Guidelines for Management of Sepsis and Septic Shock 2020, *CoE* certainty of evidence, *GRADE* Grading of Recommendations Assessment, Development, and Evaluation

**Table 2:** Characteristics of clinical questions

	Group I^a^	Group II^b^	Group III^c^
*N*	1 (5%)	10 (50%)	9 (45%)
The number of CQs where the direction (benefit or harm) of the point estimate in outcomes^d^ is consistent	0 (0%)	4 (40%)	1 (11%)
The number of outcomes of each CQ	5	3.5 (3–4)	3 (3–5)
The number of CQs where classification of the precision of each outcome			
Included “serious”	1 (100%)	8 (80%)	8 (89%)
Included “very serious”	0 (0%)	9 (90%)	8 (89%)
The proportion of outcomes with insufficient OIS in each CQ (%)	0	100 (80–100)	100 (100–100)
The number of CQs where the outcome that critical for net effect estimate^e^ could not be identified	0 (0%)	1 (10%)	1 (11%)

*CQ* clinical question, *OIS* optimal information size

^a^Certainty of evidence evaluated using net benefit approach was lower than that evaluated using the traditional method

^b^The certainty of evidence evaluated by net benefit approach was equal to that of the traditional method

^c^Certainty of evidence evaluated by net benefit approach is higher than that by the traditional method

^d^In this table, “outcomes” means outcomes to be combined for calculating net effect estimate

^e^These outcomes are as follows: 1) outcomes for which the removal of the outcome would change the classification of the precision of the net effect estimate. 2) Outcomes for which the addition of plausible increases to the effect estimate (effect estimates with lower certainty) would change the classification

This is the first study to compare the traditional method and the net benefit approach for determining the CoE. Our findings suggest that the net benefit approach tends to provide higher or equivalent CoE ratings for CQs that use dichotomous outcomes to calculate net effect estimate. The results of this study added novel insights for guideline developers to create CPGs that implement the net benefit approach concept.

These results are attributed to the net benefit approach's emphasis on critical outcomes for patients, such as death. By contrast, the traditional approach is susceptible to outcomes with lower CoEs, particularly when the point estimates for benefits or harms are inconsistent. The net benefit approach compensates for this weakness of the traditional method by considering the relative importance of each outcome.

This study has some limitations. First, most outcomes of the CQs not meeting the OIS criteria and only one CPG was evaluated. Second, one CQ showed a lower CoE in the net benefit approach than in the traditional method, so the cause could not be well discussed because of the limited sample size. Likewise, we could not discuss the possibility that the net benefit approach would lead to overestimate CoE. Further studies are required to address these limitations and evaluate the usefulness of the net benefit approach more comprehensively. Nonetheless, the significance of this report lies in providing the first comparison between traditional method and the net benefit approach to determine CoE.

We examined the disparities in CoE evaluation between the traditional and novel net benefit approach methods for 20 CQs from the J-SSCG2020. Our findings suggest that the CoE calculated using the net benefit approach tends to be equal to or higher than that calculated using the traditional method. Further rigorous investigations are necessary to identify the underlying reasons for this difference.

## Supplementary Information

**Additional file 1: Figure S1.** Steps to determining the certainty of the net effect estimate. **Figure S2.** Patterns of change in certainty of evidence (CoE) between the traditional method and the net benefit approach by clinical question. **Table S1.** The details of the included Clinical Questions.

**Additional file 2.**  Utility tool for the net benefit approach.

## Data Availability

The datasets used or analyzed in the current study are available from the corresponding author upon reasonable request.

## Abbreviations

CPG: Clinical practice guideline
GRADE: Grading of Recommendations Assessment, Development, and Evaluation
CoE: Certainty of evidence
CQ: Clinical question
J-SSCG2020: The Japanese Clinical Practice Guidelines for Management of Sepsis and Septic Shock 2020
OIS: Optimal information size
