# Supplementary material for: Comparison of certainty of evidence between the net benefit approach and the traditional GRADE method using the data of Japanese Clinical Practice Guidelines for Management of Sepsis and Septic Shock 2020
Source: J Intensive Care. 2023 Jul 17;11:33. doi: 10.1186/s40560-023-00680-5 (PMC10353182; doi:10.1186/s40560-023-00680-5)
Supplement: Supplementary file 1 — Additional file 1: Figure S1. Steps to determining the certainty of the net effect estimate. Figure S2. Patterns of change in certainty of evidence (CoE) between the traditional method and the net benefit approach by clinical question. Table S1. The details of the included Clinical Questions. [file 40560_2023_680_MOESM1_ESM.docx]

**Additional File 1**

Comparison of certainty of evidence between the net benefit approach and the traditional Grading of Recommendations Assessment, Development, and Evaluation (GRADE) method using the data of Japanese Clinical Practice Guidelines for Management of Sepsis and Septic Shock 2020

**Authors**

Takero Terayama, Hiromu Okano, Sadatoshi Kawakami, Kenichi Kano, Masaaki Sakuraya, Yoshitaka Aoki, Committee of the Japanese Clinical Practice Guidelines for the Management of Sepsis and Septic Shock 2024

**Affiliations**

1. Department of Emergency, Self-Defense Forces Central Hospital, Ikeziri 1-2-24, Setagaya, Tokyo, Japan

2. Department of Critical Care Medicine, St. Luke's International Hospital, 9-1 Akashi-cho, Chuo-ku, Tokyo 104-8560, Japan

3. Department of Anesthesiology, Chiba University Graduate School of Medicine, 1-8-1

Inohana, Chuo-Ku, Chiba, 260-8670, Japan

4. Department of Emergency Medicine, Fukui Prefectural Hospital, Yotsui 2-8-1, Fukui city, Fukui, 910-8526, Japan

5. Department of Emergency and Intensive Care Medicine, JA Hiroshima General Hospital, Jigozen 1-3-3, Hatsukaichi, Hiroshima, 738-8503, Japan

6. Department of Anesthesiology and Intensive Care Medicine, Hamamatsu University School of Medicine, Shizuoka, Japan

**Corresponding Author**

Takero Terayama, M.D., PhD

Address: Department of Emergency, Self-Defense Forces Central Hospital, Ikeziri 1-2-24, Setagaya, Tokyo, Japan

E-mail: takero.for.medical.journal@gmail.com

Telephone: +81-3-3411-0151

Fax: +81-3-3411-0151

**Table of Contents**

**Figure S1………………………………………………………………………………...3**

**Figure S2………………………………………………………………………………...4**

**Table S1………………………………………………………………………………….5**

**Figure S1. Steps to determining the certainty of the net effect estimate**


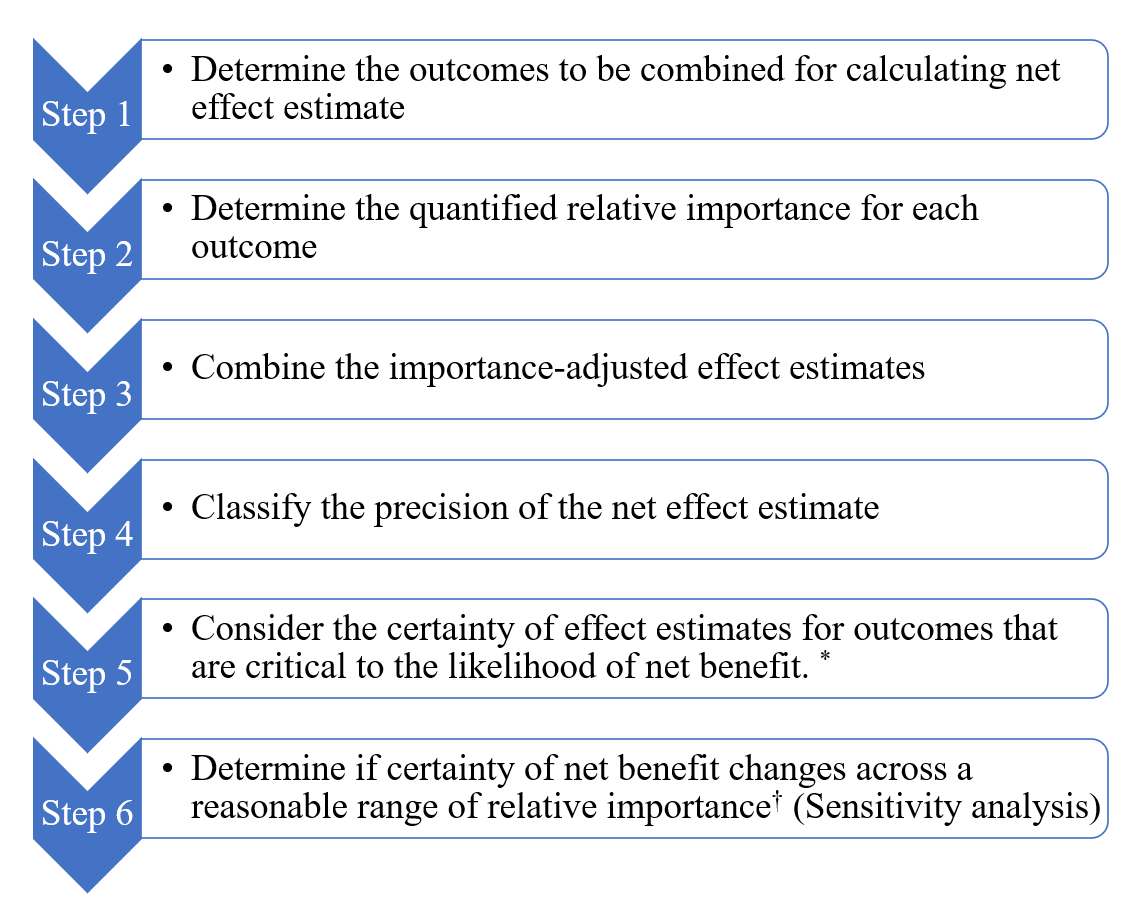


* These outcomes are:1) Outcomes for which removal of the outcome would change the classification of the precision of the net effect estimate. 2) Outcomes for which the addition of plausible increases to the effect estimate (effect estimates with lower certainty) would change the classification.

† In this study, a reasonable range was determined based on the range from the smallest to the largest value among the relative importance values determined in Step 2.

**Figure S2. Patterns of change in certainty of evidence (CoE) between the traditional method and the net benefit approach by clinical question**


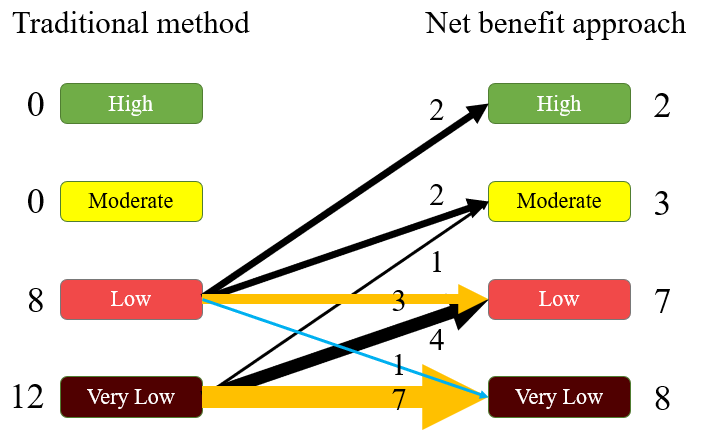


CoE, certainty of evidence.

* The classification of clinical questions was based on changes in the CoE using the net benefit approach as compared to using the traditional method: those with a decrease in the CoE (Group I), those with no change (Group II), and those with an increase (Group III).

**Table S1. The details of the included Clinical Questions**

| **Number** | **Clinical Questions** | **CoE by the traditional method** | **CoE by the net benefit approach** |
| --- | --- | --- | --- |
| 4-7 | Should continuous or extended infusion of β-lactam antibiotics be used for sepsis? | very low | very low |
| 4-8 | Should de-escalation antimicrobial therapy be used for sepsis? | low | moderate |
| 4-10 | Should relatively short-term (i.e. within 7 days) antimicrobial therapy be applied for sepsis? | very low | very low |
| 5-2-1 | Should IVIG be administered to patients with streptococcal toxic shock syndrome (STSS)? | very low | very low |
| 6-3 | Should vasopressors be used simultaneously or in the early stage (within 3 h) of initial fluid resuscitation in adult patients with sepsis? | very low | very low |
| 6-9-1 | Should noradrenaline, dopamine, or phenylephrine be used as a first-line vasopressor in adult patients with sepsis? noradrenaline vs. dopamine | very low | moderate |
| 6-9-2 | Should noradrenaline, dopamine, or phenylephrine be used as a first-line vasopressor in adult patients with sepsis? noradrenaline vs. phenylephrine | very low | low |
| 7-2 | Should hydrocortisone and fludrocortisone be administered to patients with septic shock who do not respond to initial fluid resuscitation and vasopressors? | low | high |
| 7-3 | Should corticosteroids (hydrocortisone) be administered to patients with sepsis without shock? | low | low |
| 11-1 | Should furosemide be used to prevent or treat septic AKI? | low | low |
| 11-2 | Should atrial natriuretic peptide (ANP) be used to prevent or treat septic AKI? | very low | very low |
| 11-3 | Should dopamine be used to prevent or treat septic AKI? | very low | very low |
| 11-5-2 | Should RRT be initiated early for septic AKI (Stage 3 vs. absolute indications) | very low | low |
| 11-6 | Should a large RRT dose be delivered for septic AKI? | low | low |
| 12-5 | Should parenteral nutrition be combined with enteral nutrition in septic patients? | very low | low |
| 15-3 | Should antithrombin replacement therapy be administered in sepsis-associated DIC? | low | high |
| 15-4 | Should heparin or heparin analogs be administered in sepsis-associated DIC? | very low | low |
| 15-5 | Should recombinant thrombomodulin be administered to patients with sepsis-associated DIC? | low | moderate |
| 15-6 | Should protease inhibitors be administered to patients with sepsis-associated DIC? | very low | very low |
| 22-1 | Should antiulcer drugs be administered to septic patients to prevent gastrointestinal bleeding? | low | very low |

CoE, certainty of evidence; AKI, acute kidney insufficiency; RRT, renal replacement therapy; DIC, disseminated intravascular coagulation.
